# Congenital Short QT Syndrome

**Published:** 2004-04-01

**Authors:** Charles Antzelevitch, Johnson Francis

**Affiliations:** * Executive Director and Director of Research, Gordon K. Moe Scholar, Professor of Pharmacology, Masonic Medical Research Laboratory; ¶ Associate Professor of Cardiology, Medical College Calicut, Kerala, India

Long QT intervals in the ECG have long been associated with sudden cardiac death.  The congenital long QT syndrome was first described in individuals with structurally normal hearts in 1957 [1].  Little was known about the significance of a short QT interval. In 1993, after analyzing 6693 consecutive Holter recordings Algra et al concluded that an increased risk of sudden death was present not only in patients with long QT interval, but also in patients with short QT interval (< 400 ms) [2].  Because this was a retrospective analysis, further evaluation of the data was not possible.

It was not until 2000 that a short-QT syndrome (SQTS) was proposed as a new  inherited clinical syndrome by Gussak et al [3].  The initial report was of two siblings and their mother all of whom displayed persistently short QT interval. The youngest was a 17 year old female presenting with several episodes of paroxysmal atrial fibrillation requiring electrical cardioversion [3]. Her QT interval measured 280 msec at a heart rate of 69.  Her 21 year old brother displayed a QT interval of 272 msec at a heart rate of 58, whereas the 51 year old mother showed a QT of 260 msec at a heart rate of 74. The authors also noted similar ECG findings in another unrelated 37 year old patient associated with sudden cardiac death.

A review on the subject including a discussion of proposed mechanisms appeared in 2002 [4].  The review highlighted the lack of rate-dependence of QT interval in cases in which QT abbreviation was constant and the negative correlation of QT with RR in cases in which an abnormally short QT was evident only at bradycardic rates.  Among the principal gene candidates proposed to underlie these syndromes were gain of function mutations of I_Kr_, I_Ks_, I_K-ACh_ and I_K-ATP_ [4] (Figure 1).   I_K-ACh_ gain of function or other means by which the influence of the parasympathetic nervous system could be exaggerated was considered as the most likely mechanisms to explain the deceleration-dependent variant of the short QT syndrome.

The familial nature of this sudden death syndrome was confirmed by Gaita et al in 2003 [5]. They reported a study of six patients from two different families, with syncope, palpitations, resuscitated cardiac arrest and a positive family history for sudden cardiac death. The QT intervals never exceeded 280 msec or a QTc 300 msec. There was no evidence of structural heart disease in any family member. Electrophysiological evaluation yielded short atrial and ventricular refractory periods in all four patients who underwent testing. Three of them were also found to have increased vulnerability to ventricular fibrillation.  Four patients received an automatic defibrillator (ICD) [5].

Factors that abbreviate the QT interval, including tachycardia, hyperthermia, hypercalcemia and digoxin should naturally be excluded before arriving at a diagnosis of congenital short QT syndrome. The mechanism of arrhythmogenesis in some cases of digitalis toxicity could be similar to those in congenital short QT syndrome [6].

The first gene responsible for the short QT syndrome was reported by Brugada et al in January of 2004 [7].  A candidate gene approach was used to screen for a causative mutation in the two families previously reported by Gaita et al [5]. Using direct sequencing techniques, two different missense mutations were uncovered in the two families, resulting in the same amino acid substitution in HERG (KCNH2), the gene encoding for the rapidly activating delayed rectifier channel, I_Kr_ [7]. The substitution of lysine for asparagines at position 588 of KCNH2, was found to cause a loss of the normal rectification of the current at plateau voltages, thus resulting in a large increase of I_Kr_ during the action potential plateau, leading to marked abbreviation of the action potential. The short QT syndrome is the first disease to be linked to a gain of function of I_Kr_. A third short QT family was not associated with a mutation in KCNH2, pointing to genetic heterogeneity of the disease. Interestingly, the N588K mutant channel proved to be insensitive to I_Kr_ blockers such as d-sotalol both in the clinic and in heterologous expression systems. Quinidine, by virtue of its greater affinity for the open state of the channel, its additional I_Ks_ blocking actions and anticholinergic activity was found to be more effective in reducing the repolarizing forces of the ventricle and prolonging the action potential [8].

It is noteworthy that substitution of aspartic acid for asparagine in the same position of HERG (N588D) has been linked to the LQT2 form of the long QT syndrome secondary to a loss of function of IKr [9].  This substitution leads to replacement of a neutral amino acid with a negatively charged one, whereas the N588K mutation responsible for short QT, secondary to gain of function of I_Kr_, replaces a neutral amino acid with a positively charged one.

Bellocq et al recently linked a second gene to the syndrome.  A missense mutation in KCNQ1 (KvLQT1) giving rise to a gain of function in I_Ks_, the slowly activating delayed rectifier, was identified in a 70 year old male presenting with idiopathic ventricular fibrillation and short QT intervals in the ECG. Functional studies of the V307L KCNQ1 mutant revealed a -20 mV shift of the half-activation potential and an acceleration of the activation kinetics leading to an increase in I_Ks_ [10].

A distinctive electrocardiographic feature of the short QT syndrome is the appearance of tall peaked T waves, similar to those encountered with hyperkalemia. ICD implantation is currently the treatment of choice for symptomatic patients with short QT syndrome and a family history of sudden cardiac death. Despite normal sensing behavior during device testing, inappropriate shocks due to T wave over sensing has been reported. This is due to the detection of short-coupled and prominent T waves [11]. The problem was corrected by reprogramming to lower sensitivity and decay delay. More specific diagnostic criteria will need to be developed as additional information about this syndrome becomes available in the coming months and years.

## Figures and Tables

**Figure 1:**
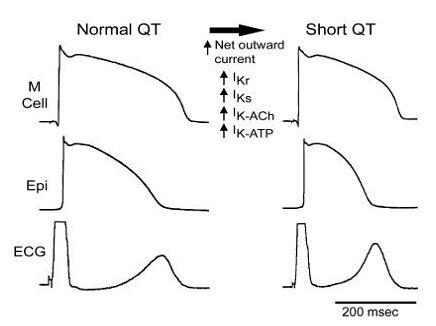
Schematic diagram illustrating cellular changes attending the abbreviation of the Short QT Syndrome secondary to an increase in net outward repolarizing current. Epi=epicardium; ECG=electrocardiogram.

## References

[R1] Jervell A, Lange-Nielsen F (1957). Congenital deaf-mutism, functional heart disease with prolongation of the QT interval and sudden death. Am Heart J.

[R2] Algra A, Tijssen JGP, Roelandt JRTC (1993). QT interval variables from 24-Hour electrocardiography and the 2- Year risk of sudden death. Br Heart J.

[R3] Gussak I, Brugada P, Brugada J (2000). Idiopathic short QT interval: a new clinical syndrome?. Cardiology.

[R4] Gussak I, Brugada P, Brugada J (2002). ECG phenomenon of idiopathic and paradoxical short QT intervals. Cardiac Electrophysiology Review.

[R5] Gaita F, Giustetto C, Bianchi F (2003). Short QT Syndrome: a familial cause of sudden death. Circulation.

[R6] Cheng TO (2004). Digitalis administration: an underappreciated but common cause of short QT interval. Circulation.

[R7] Brugada  R, Hong K, Dumaine R (2004). Sudden Death associated with Short QT Syndrome linked to Mutations in HERG. Circulation.

[R8] Cordeiro JM, Brugada R, Hong K (2004). Short QT syndrome mutation in HERG abolishes inactivation (Abstract). Biophysical Journal.

[R9] Splawski I, Shen J, Timothy KW (1998). Genomic structure of three long QT syndrome genes: KVLQT1, HERG, and KCNE1. Genomics.

[R10]  Bellocq C, van Ginneken A, Bezzina CR (2004). A molecular and pathophysiological substrate for the short QT interval syndrome. Circulation.

[R11] Schimpf R, Wolpert C, Bianchi F (2003). Congenital short QT syndrome and implantable cardioverter defibrillator treatment: inherent risk for inappropriate shock delivery. J Cardiovasc Electrophysiol.

